# Ovarian cancer cell invasiveness is associated with discordant exosomal sequestration of Let-7 miRNA and miR-200

**DOI:** 10.1186/1479-5876-12-4

**Published:** 2014-01-06

**Authors:** Miharu Kobayashi, Carlos Salomon, Jorge Tapia, Sebastian E Illanes, Murray D Mitchell, Gregory E Rice

**Affiliations:** 1 Centre for Clinical Diagnostics, Royal Brisbane and Women’s Hospital, University of Queensland Centre for Clinical Research, Building 71/918, Herston, Queensland 4029, Australia; 2 Department of Obstetrics and Gynaecology, Faculty of Medicine, Universidad de los Andes, Santiago, Chile

**Keywords:** Ovarian cancer, Exosomes, microRNA, Biomarkers, Invasion

## Abstract

**Background:**

The role of exosomes in the pathogenesis and metastatic spread of cancer remains to be fully elucidated. Recent studies support the hypothesis that the release of exosomes from cells modifies local extracellular conditions to promote cell growth and neovascularisation. In addition, exosomes may modify the phenotype of parent and/or target cell. For example, sequestration of signaling mediators into exosomes may reduce their intracellular bioavailability to the parent cell thereby altering cell phenotype and metastatic potential. The fusion of released exosomes with target cell and delivery may also modify cell function and activity. In this study, to further elucidate the role of exosomes in ovarian cancer, the release of exosomes from two ovarian cancer cell lines of different invasive capacity and their miRNA content of exosomes were compared. The hypothesis to be tested was that ovarian cancer cell invasiveness is associated with altered release of exosomes and discordant exosomal sequestration of miRNA.

**Methods:**

High (SKOV-3) and low (OVCAR-3) invasive ovarian cancer cell lines were used to characterize their exosome release. SKOV-3 and OVCAR-3 cells were cultured (DMEM, 20% exosome-free FBS) under an atmosphere of 8% O_2_ for 24 hours. Cell-conditioned media were collected and exosomes were isolated by differential and buoyant density centrifugation and characterised by Western blot (CD63 and CD9). Exosomal microRNA (let-7a-f and miR-200a-c) content was established by real-time PCR.

**Results:**

Exosomes were identified with by the presence of typical cup-shaped spherical vesicle and the expression of exosome markers: CD63, CD9. SKOV-3 cells released 2.7-fold more exosomes (1.22 ± 0.11 μg/10^6^ cells) compared to OVCAR-3 (0.44 ± 0.05 μg/10^6^ cells). The let-7 family miRNA transcripts were identified in both ovarian cancer cell lines and their exosomes. The let-7 family transcripts were more abundant in OVCAR-3 cell than SKOV-3 cells. In contrast, let-7 family transcripts were more abundant in exosomes from SKOV-3 than OVCAR-3. miR-200 family transcripts were only identified in OVCAR-3 cells and their exosomes.

**Conclusions:**

The data obtained in this study are consistent with the hypothesis that the releases of exosomes varies significantly between ovarian cancer cell lines and correlates with their invasive potential.

## Background

Ovarian cancer continues to be the leading causes of death among women with gynaecological cancer with 225,500 new cases reported each year, causing over 140,200 deaths annually worldwide [1]. Although ovarian cancer accounts for 3% of all cancer incidents, 6% of cancer-related death is caused by ovarian cancer, making it the fifth leading cause of cancer mortality in women [2]. While progress has been achieved in our understanding of aetiology and risk factor for ovarian cancer research, there has been little improvement in survival rates [2]. Ovarian cancer continues to be a challenging public health issue.

The keystone to improving health outcomes remains the timely and accurate diagnosis of the predisposition to, or early detection of disease. Early detection of disease risk and onset is the first step in implementing efficacious treatment and improving patient outcome. In the context of ovarian cancer, despite recent progress in chemotherapeutic treatments, the diagnosis of late stage disease is associated with a five-year survival rate of ~30%. In contrast, when ovarian cancer is identified at an early stage, five year survival increases to ~90% [3]. Thus, the development of more accurate and earlier detection tests for this disease is undoubtedly the number one priority for achieving long- term reduction of mortality from ovarian cancer. To date, such tests have not been developed and the use of traditional biomarker approaches and screening methods have failed to deliver significant clinical outcomes. New approaches to the development of in vitro diagnostics that deliver greater diagnostic sensitivity are required.

Recent studies highlight the putative utility of tissue-specific nanovesicles (*e.g.* exosomes) in the diagnosis of disease onset and treatment monitoring [4,5]. To date, there are only limited data defining changes in the release, role and diagnostic utility of ovarian cancer-derived exosomes.

Exosomes are small (40–90 *nm*) membrane vesicles of endocytic origin. The biogenesis of exosomes is initiated by the inward budding of multivescular bodies (MVB), encapsulating cellular proteins and RNA molecules to form internal vesicles. Exosomes are released from several cell types including cancer cells into the peripheral circulation and are detected in biofluids such as plasma, saliva, urine and breast milk [6,7].

Tumour-derived exosomes may affect cancer progression in several ways. Tumour-derived exosomes have the ability to propagate oncogenic activity among tumour cells.

Exosomes are not only mediators of cell-to-cell communication but also represent an attractive source of cancer biomarkers due to the following features: exosomes are *(i)* actively released from living tumour cells; *(ii)* convey information about tumour state; *(iii)* easily obtained from biofluids; easily isolated from high-abundance proteins that confound biomarker discovery; and *(iv)* are high stability. Most importantly, exosomes are being secreted from living tumour cells and are distinct from apoptotic cell-derived microvesicles [8]. As exosomes contain cellular protein and RNA molecules in cell type-specific manner, they may provide extensive information about the signature of the tumour [9]. Exosomes have been reported to express a diverse range of cell surface receptors, proteins (including, heat shock proteins, cytoskeletal proteins, adhesion molecules, membrane transport and fusion proteins) and miRNA with the potential to affect the acute and long-term function of the cells with which they interact. miRNA is a class of small (approximately 22 nt long), non-coding RNAs that negatively regulate gene expression by binding to the 3′ untranslated region of target mRNAs [10,11]. Once the miRNA is bound, the target messenger RNA (mRNA) is either cleaved for degradation or its translation is inhibited [12]. miRNAs are evolutionary conserved across species, reinforcing the vast influence of miRNAs on essential biological processes such as differentiation, proliferation, apoptosis [10,12,13]. Deregulation of these miRNAs will not only impact normal physiological processes but also implicated in diseases including cancer. Previous studies have established the significant difference in ovarian cancer miRNA profiles, reinforcing miRNA as a promising cancer biomarker, most studies, however, have examined the miRNA profile of tumour tissues. The collection of tissue samples is an invasive procedure and unsuitable for a diagnostic and screening tests. The utility of cell-free miRNA in biofluids has been investigated as a source of cancer biomarkers. Although this approach overcomes the issue of sample collection, the question remains on how miRNAs are released and avoid degradation. Currently, limited data are available on the mechanism of free miRNA release. The origin of these miRNAs remains unclear and they may be released from apoptotic cells. If this is the case, free miRNAs may not be a useful indicator of tumours state and/or progression.

The let-7 family of miRNAs comprises 10 mature isoforms and is important in development and cell fate control. They are initially expressed as primary (pri)miRNA in the form of a hairpin loop and the base is removed by RNaseII enzyme, Drosha, to form pre-miRNA. PremiRNA is then exported from nucleus to cytoplasm where another RNaseII enzyme, Dicer, cleaves the loop region to produce the mature miRNA. The mature miRNA is incorporated into the RNA induced silencing complex as a guide for target mRNA [14]. Let-7 expression is deregulated in aggressive high-grade ovarian cancer [15]. In addition, miR-200 family (i.e. miR-200a, miR-200b and miR-200c) has been associated with ovarian cancer progression [16]. We hypotheses that (i) the release of exosomes from ovarian cancer cell lines is responsive to changes with invasiveness capacity; and (ii) exosomal miRNA content is cell type specific.

The hypothesis to be tested was that ovarian cancer cell invasiveness is associated with altered release of exosomes and discordant exosomal sequestration of miRNA. Two ovarian cancer cell lines of epithelial cell origin but with different invasive potentials (SKOV-3 and OVCAR-3) were used to test the hypotheses.

## Methods

### Ovarian cancer cell lines

This study was approved by the Human Research Ethics Committees of the Royal Brisbane and Women’s Hospital, and the University of Queensland (HREC/09/QRBW/14). All experimental procedures were conducted within an ISO17025 accredited (National Association of Testing Authorities, Australia) research facility. All data were recorded within a 21 CRF part 11 compliant electronic laboratory notebook (Irisnote, Redwood City, CA, USA). The human ovarian cancer cell lines OVCAR-3 and SKOV-3 (with more invasive capacity than OVCAR-3) [17] were obtained from the American Type Cell Collection and were cultured in RPMI media (Life technologies, USA) supplemented with 10% fetal bovine serum (FBS; PAA, Australia). Cell cultures were incubated at 37°C in 8% O_2_ and 5% CO_2_ atmosphere. Cell confluence and morphology were routinely checked, cells washed with phosphate buffered saline (PBS; Life technologies, USA) and fresh growth media was added every two to three days. Cells were subcultured with dissociation media, TrypLE™ Express (Life technologies, USA) and cellular viability was determined by Trypan Blue exclusion and Countess® Automated cell counter (Life Technologies, USA).

### Invasion assay of OVCAR-3 and SKOV-3

Cell invasion rates were established using our previously published method [18,19]. Using a real-time cell imaging system (IncuCyte™ (Essen BioScience, Michigan, USA). In brief, 96-well plates were coated with a thin layer of collagen by transferring 300 μg/ml of collagen type I (Life Technologies™, Carlsbad, CA) and incubating at 37°C for 30 min. OVCAR-3 and SKOV-3 (2 × 10^4^ cells per well) were grown to confluence in complete growth media. Cell-free zones were created by generating a wound with a 96-Well WoundMaker™ (Essen BioScience, Michigan, USA). The cells were overlaid with 3 mg/ml collagen type I (Life Technologies™, Carlsbad, CA) and incubated at 37°C for 30 min to create a 3D matrix. Complete growth media was added on top of the layer of collagen. Cells were imaged automatically every 3 h over a time period of 48 h. The images were processed by the IncuCyte™ software package (Essen BioScience, Michigan, USA) to measure cell invasion by obtaining the Relative Wound Density (RWD, as defined by custom algorithms within the IncuCyte™ software package). These users informed algorithms identify the wound region and provide visual representations of the segmentation parameters. Image collection was created using three to five representative phase contrast images.

### Exosome isolation from cell-cultured media

Prior to exosome isolation, cell cultures grown above 70% confluence were washed with PBS and exosome-free media was added. Exosome-free media composed of RPMI media supplemented with 20% exosome-depleted FBS and 5% antibiotic-antimycotic (Life technologies, USA). Exosomes were removed from FBS by ultracentrifuging at 100,000 × *g* for 21 h at 4°C and filtering the resulting supernatant with 0.22 μm filter (Millipore, Massachusetts, USA). The cells were incubated in exosome-free media for 24 h at 37°C in 8% O_2_ and 5% CO_2_ atmosphere. The following day, exosomes were isolated from cell-cultured media by differential ultracentrifugation as previously described [18,19] Cell-conditioned cultured media was centrifuged successively at increasing speed: (*i*) 300 × *g* for 5 min 4°C, (*ii*) 1200 × *g* for 10 min 4°C, (*iii*) 10,000 × *g* for 30 min 4°C, (*iv*) 100,000 × *g* for 75 min 4°C. After each step, the pellet was discarded and the supernatant was quantified and used for the following step. The resulting pellet after the fourth step was resuspended in PBS and washed at 100,000 × *g* for 70 min 4°C to remove any contaminating proteins. The pellet was resuspended in PBS, layered on a cushion of 30% (w/v) sucrose and centrifuged at 110,000 g for 75 min for purification. The fraction containing OVCAR-3 and SKOV-3 exosomes (~3.5 ml, 1.127 density using OPTi digital refractometer (Bellingham^+^Stanley Inc., Lawrenceville, GA, USA) was recovered with an 18-G needle and diluted in PBS, and then centrifuged at 110 000 × g of 70 min.

### Cell count and viability

Cells were washed with PBS and dissociated with TrypLE™ Express (Life technologies, USA). The number and viability of cells were determined to further calculate the amount of exosome release from one million cells. Collected cells were incubated with Trypan Blue, cell number and viability were assessed using a Countess® automated cell counter (Invitrogen™, USA).

### Exosomal and cellular protein quantification

Amount of exosomes secreted were estimated by measuring the total protein present within exosomes by the BIO-RAD DC™ Protein Assay (USA). Exosome samples were prepared by adding RIPA buffer directly to exosomes suspended in PBS and sonicated at 40°C for 15 s three times to lyse exosome membrane and solubilise the proteins. Bovine serum albumin (BSA) diluted in RIPA and PBS mixture (1:1) were prepared as protein standards at 0, 200, 400, 600, 800, 1000, 1500 μg/mL. Standards and samples (prepared exosomes and cell lysate) were transferred to 96-well plates and procedures outlined by the manufacture were followed. In brief, alkaline copper tartrate solution (BIO-RAD, USA) and dilute Folin Reagent (BIO-RAD, USA) were added to the samples and incubated for 15 min. The absorbance was read at 750 *nm* with Paradigm Detection Platform (Beckman Coulter, USA).

### Western Blot

Exosome markers were identified by Western blot according to our previously published methods [18]. In brief, 10 μg of exosomal proteins were separated on NuPAGE 4-12% Bis-Tris Gel (Life Technologies, USA). Separated proteins were transferred to Immobilon-®FL polyvinylidene difluoride membrane (Millipore, Billerica, MA, USA) in transfer buffer (5% NuPAGE® Transfer buffer, 20% methanol) for 1 hour at 100 V. The membrane was washed in wash buffer (PBS TWEEN 0.1%) three times for 10 min and blocked with 5% skim milk in PBS TWEEN (0.1%) for an hour at room temperature under agitation. The blocked membrane was further probed for previously identified exosome-specific markers: primary mouse monoclonal anti-CD63 (1:1000, Abcam®) or anti-CD9 (1:1000, Abcam®). This was done by incubating the membrane in primary antibody diluted in 5% skim milk in PBS TWEEN (0.1%) at 4°C overnight on the laboratory rocker. After an overnight incubation, the membrane was washed with wash buffer and exposed to secondary antibody, donkey anti mouse IgG-HRP (1:1000, Santa Cruz Biotechnology) diluted in 5% skim milk in PBS TWEEN (0.1%) for 1 h at room temperature under agitation. The membrane was washed 3 times for 10 min in wash buffer. Bound antibodies were detected by enhanced chemiluminescence (peroxide buffer and enhancer buffer, Thermo Scientific) and visualised with the SRX-101A Tabletop Processor (Konica Minolta, Ramsey, NJ, USA).

### Transmission electron microscopy

The exosome fraction isolated by differential and buoyant density gradient centrifugation was assessed by transmission electron microscopy as described [18,19]. Exosome pellets were fixed in 3% (w/v) glutaraldehyde and 2% paraformaldehyde in cacodylate buffer, pH 7.3 and the sample was then applied to a continuous carbon grid and negatively stained with 2% uranyl acetate. The samples were examined in an FEI Tecnai 12 transmission electron microscope (FEI™, Hillsboro, Oregon, USA).

### Nanoparticle tracking analysis

The size distribution of exosome preparations was analysed using a NanoSight LM10 system (NanoSight, Amesbury, UK) according to the manufacturer’s instructions. Exosome samples isolated from OVCAR-3 and SKOV-3 cells were diluted in PBS 1X 1:3 and 1:6, respectively before the analysis. The instrument measured the rate of Brownian motion of nanoparticles [20].

### Quantification of ovarian cancer cell-derived exosome

Exosome concentration was determined using the total exosomal CD63 protein measured by ELISA (ExoELISA™, System Biosciences, Mountain View, CA) according to the manufacturer’s instructions. Quantitative results (number of exosome particles) were obtained using a exosome protein standard curve calibrated by NanoSight instrument provide by ExoELISA™ kit.

### miRNA isolation

Ambion mirVana PARIS Kit (Invitrogen, USA) was used to extract cellular and exosomal total RNA from OVCAR-3 and SKOV-3 by following the manufacturer’s procedure. Cell samples were prepared by trypsinising the cells and centrifuging at 300 × g for 5 min to remove the supernatant. Samples are first lysed by adding cell disruption buffer and vortexed or pipetted vigorously. Denaturing solution was added to samples and incubated on ice for 5 min. The first two steps stabilize RNA and inactivate RNases. The lysate is then subjected to Acid-Phenol:Chloroform extraction by adding Acid-Phenol:Chloroform, vortexed and centrifuged at 10,000 × g for 5 min. Recovery of the aqueous phase obtains semi-pure RNA samples, removing most of the other cellular components. 100% ethanol was mixed and passed through a filter cartilage. The filter was washed three times and the RNA was eluted with nuclease-free water.

### Real-time PCR

Reverse transcription was performed using the miScript Reverse Transcription Kit (QIAGEN, Valencia, CA, USA) in a total volume of 20 μl. cDNA was synthesised from the maximum volume of OVCAR-3 and SKOV-3 exosomal RNA (12 μl) using the BIO-RAD T100™ Thermal Cycler (USA) running for 60 min at 37°C, 5 min for 95°C and 60 min for 37°C. As the control, RNase-free water was added as the RNA template. Real-time PCR was performed with miScript SYBR Green Kit (QIAGEN, Valencia, CA, USA). Forward primers (miScript primer assays, QIAGEN, Valencia, CA, USA) designed to detect the following mature miRNAs were used: let-7 family (Hs_let-7a_2, Hs_let-7b_1, Hs_let-7c-1, Hs_let-7d_2, Hs_let-7e_3, and Hs_let-7f_1) and miR-200 family (Hs_miR-200a_1, Hs_miR-200b_3, Hs_miR-200c_1). The universal primer included in the kit was used as the reverse primer. The reactions were performed in triplicate using the BIO-RAD iQ™5 Multicolor Real-Time PCR Detection System (USA) with the following conditions: 94°C for 3 min, 35 amplification cycles of 94°C for 45 s, 55°C for 30 s and 72°C for 30 s, 72°C for 10 min, 12°C for ∞ min. Let-7 miRNA and miR-200 expression was normalized (∆C_T_) to the recommended housekeeping gene, human RNU6-2 (RNU6B) where ∆C_T_ = C_T*LET-7* or *miR-200*_ - C_T*RNU6B*_. The data are presented as 2^-∆CT^. miRNA transcript expression from the same amount of RNA (Ambion mirVana PARIS Kit) was measured. RNU6-2 expression was consistent in all the samples both within and across experimental conditions. No statistically significant differences (p > 0.05) in the expression of RNU6-2 between exosomes and/or cells samples measured by Standard Deviation of C_T_ were identified.

### Statistical analysis

All studies conducted were treatment – control comparisons, with at least 3 independent experiments (*n* value) performed. Data are presented as mean ± SEM (*n* value). Comparisons between two or more groups were performed by means of unpaired Student’s *t*-test and analysis of variance (ANOVA), respectively. Statistical significance was defined at least *p < 0.05* determined using the GraphPad Prism software, *Inc.*

## Results

### Characterisation of human epithelial ovarian cancer cell lines

**Figure 1:**
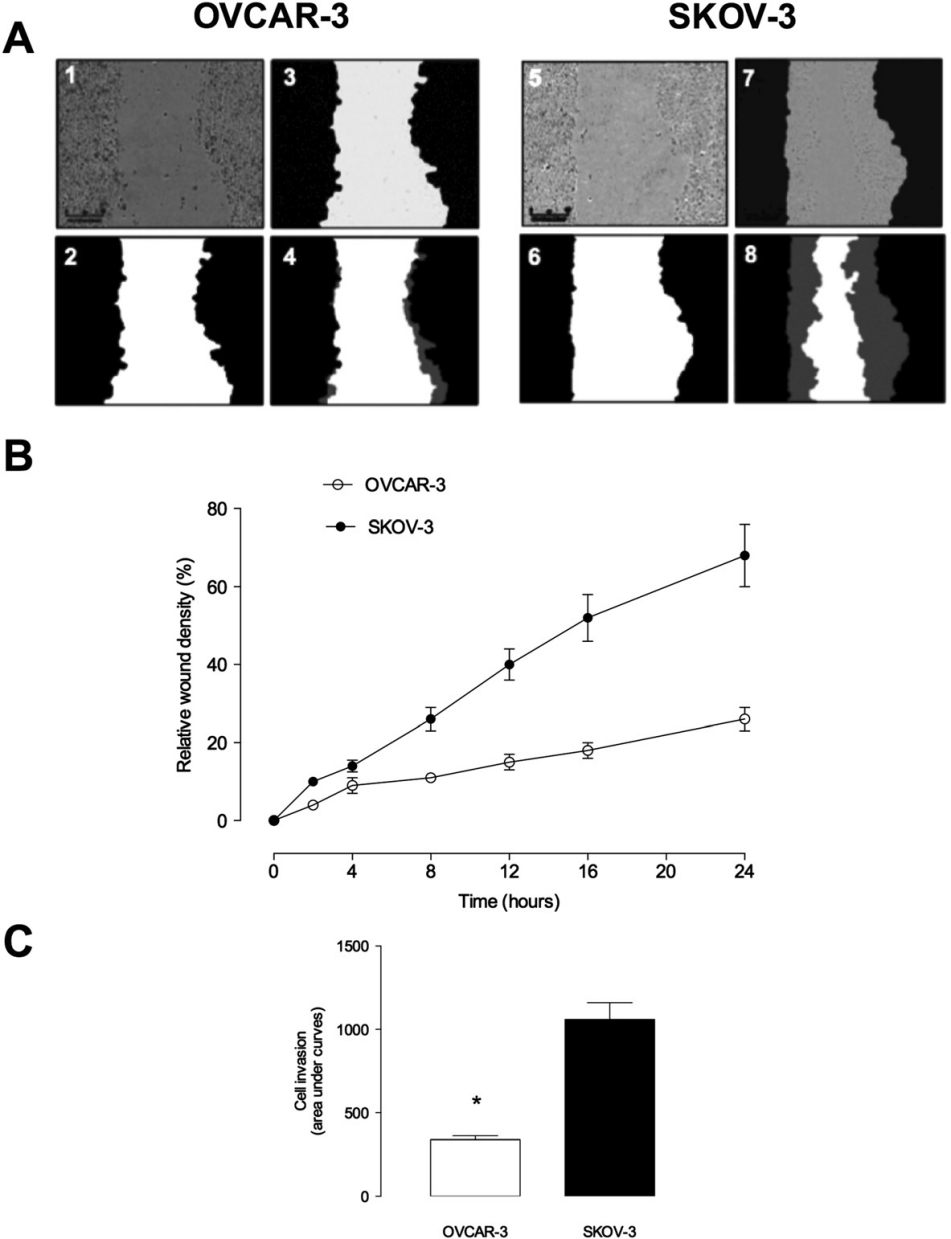
**Ovarian cancer cell invasion.** OVCAR-3 and SKOV-3 cells were grown to confluence in complete media. A wound was made using 96 well WoundMaker and then overlaid to form a 3D matrix-gel. Invasion assay was preformed with OVCAR-3 and SKOV-3 cells in complete media. Cells were imaged with IncuCyte™ (Essen BioSciences, USA) every three hours for 24 h. (see Methods). **(A)** Representative images of OVCAR-3 (A1-A4) and SKOV-3 (A5-A8) cell invasion. A1 and A5: phase contrast micrograph image immediately after wounding; A2 and A6: graphical representation showing the calculation of initial wound width; A3 and A7: cells images show 24 hours later; A4 and A8: graphical representation of cell invasion at 24 hours of the experiment. The grey region denotes the area of the initial wound covered by advancing cells. **(B)** The time course of OVCAR-3 and SKOV-3 invasion. **(C)** Area under curves analysis from **B**. Data represented as mean ± SEM (*n =* 6). In **C**, *P < 0.001 versus SKOV-3.

Initially, studies were conducted to confirm the invasiveness of the two ovarian cancer cell lines (OVCAR-3 and SKOV-3 cells) used in these studies. Figure 1A presents photomicrographs of wound closure over 24 h incubation for both cell lines and the percent change in relative wound density over time (Figure 1B). Figure 1C presents cell invasion expressed as area under the curve (AUC) of the change in relative wound density overtime. AUC for SKOV-3 cell was 3.2 fold greater than the AUC for OVCAR-3 cells (p < 0.0001). The data obtained are consistent with SKOV-3 cells being significantly more invasive than OVCAR-3 cells.

### Characterisation of ovarian cancer cell line-derived exosomes

**Figure 2:**
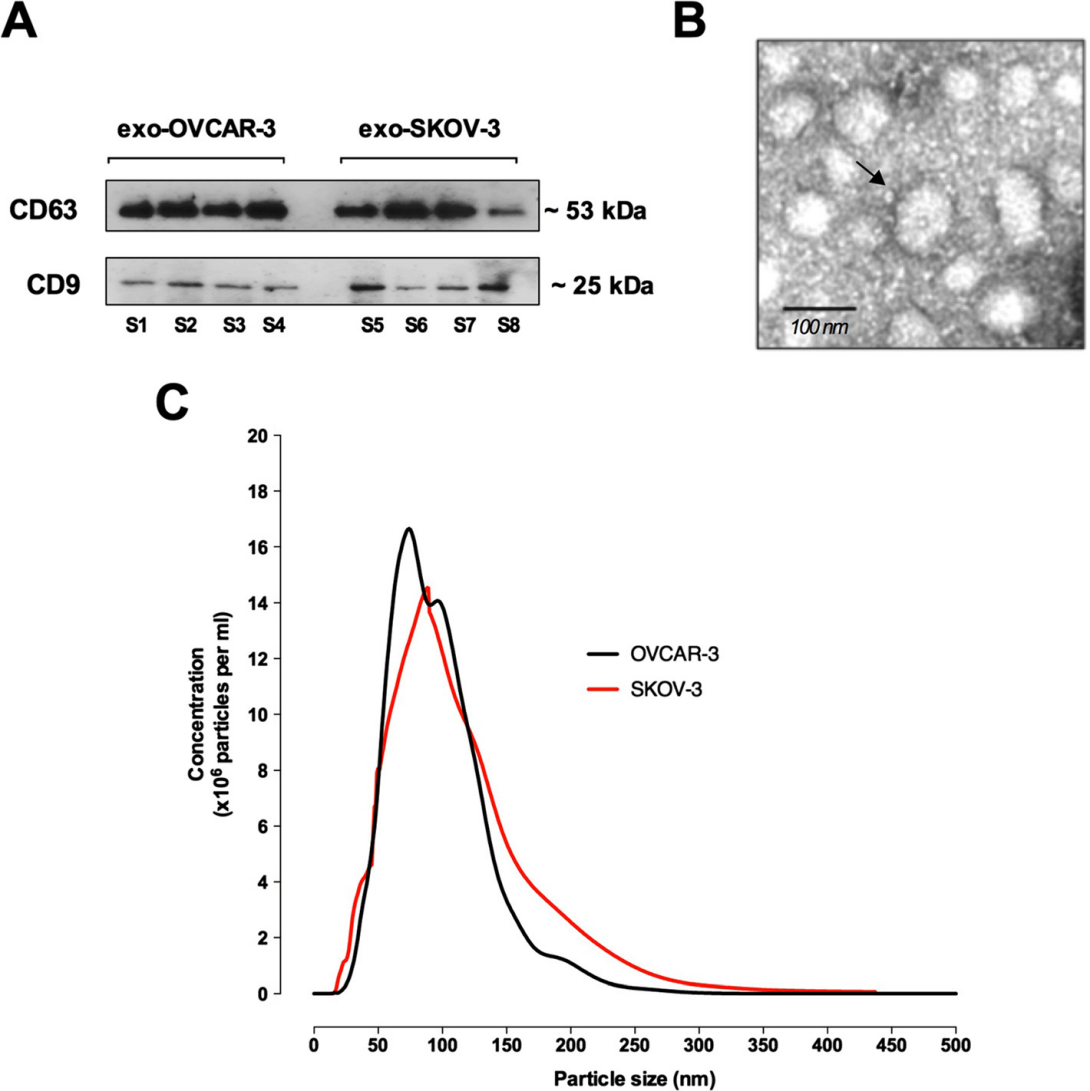
**Characterisation of exosomes from ovarian cancer cell lines. (A)** Representative image of western blot for the presence of CD63 and CD9. S1-S4 and S5-S6 represent different exosomes isolation from OVCAR-3 and SKOV-3 cells, respectively. **(B)** Representative electron micrograph of exosomes isolated from OVCAR-3 and SKOV-3 cells. **(C)** Nanosight measurement of particle-size distribution in preparation from OVAR-3 (black) and SKOV-3 (red) exosomes (see Methods). In B, *Scale bar* 100 nm.

Exosomes derived from OVCAR-3 and SKOV-3 were isolated from cell-conditioned cultured media by differential and buoyant density centrifugation. The presence of exosomal markers, CD63 and CD9 in exosomes isolated from both cell lines was confirmed by Western blot (Figure 2A). The exosomal particulate fraction isolated from OVCAR-3 and SKOV-3 were examined under transmission electron microscopy. Exosomes were identified as small vesicles between 40–100 nm in a cup-shaped form from both cell lines (Figure 2B). Finally, we compared the size distribution of exosome preparations from OVAR-3 and SKOV-3 cells supernatants using a nanoparticle tracking analysis (NanoSight). OVAR-3 and SKOV-3 exosomes were characterised by a particle size range of 50–130 nm (Figure 2C).

#### Exosome release from OVCAR-3 and SKOV-3 cells

**Figure 3:**
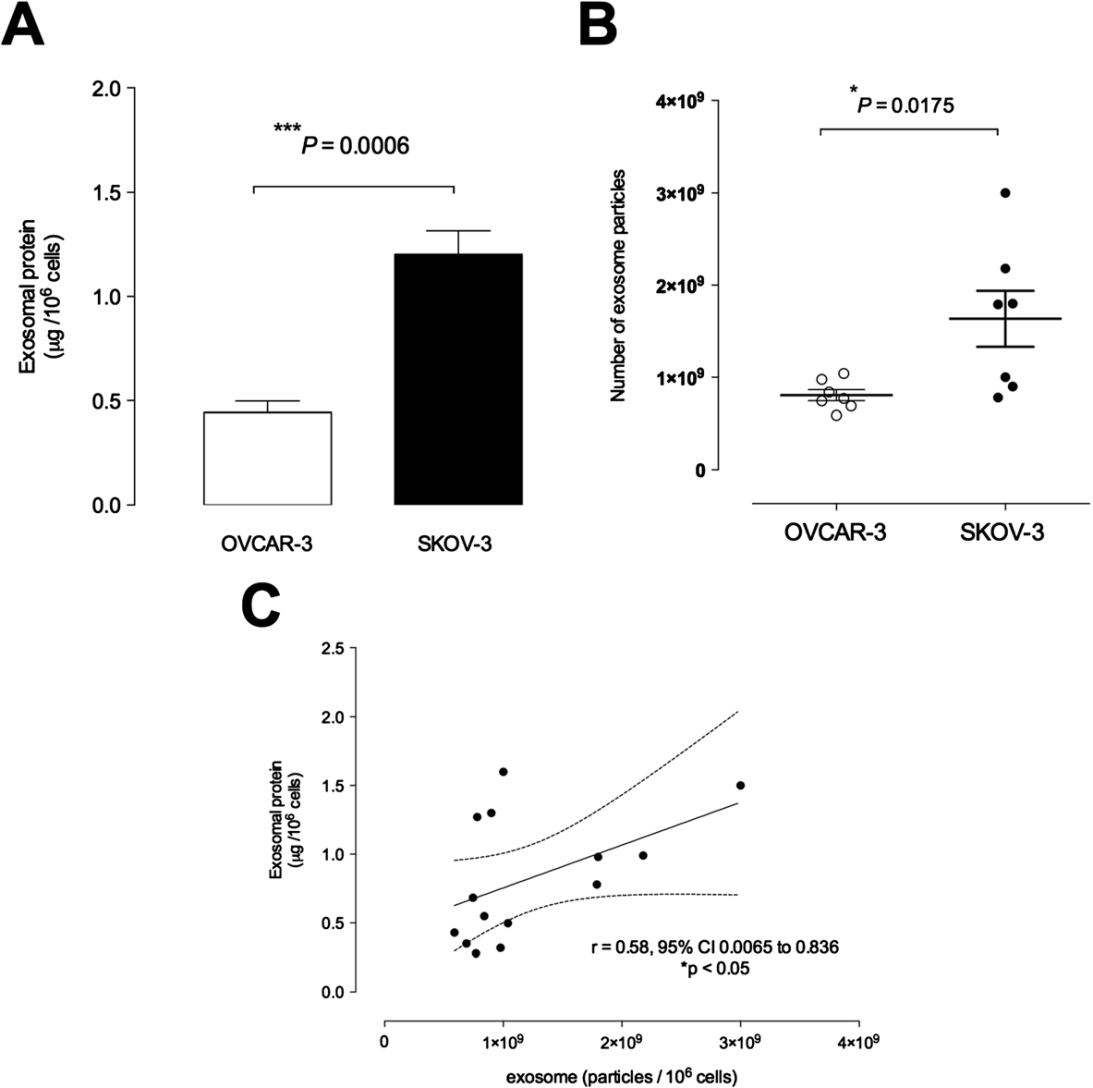
**Exosome releases from ovarian cancer cell lines.** Exosomes were quantified in culture media of ovarian cancer cell lines using an ELISA kit (see Methods). **(A)** Levels of exosome as presented as protein concentration from1x10^6^ OVCAR-3 (white bar) and SKOV-3 cells (black bar). **(B)** Quantification of number of exosome particles from OVCAR-3 (white circles) and SKOV-3 cells (black circles) per 10^6^ cells. **(C)** Relationship between number of exosome particles and exosomal protein concentration from OVCAR-3 and SKOV-3 cells. Fitted linear regression line with 95% confidence intervals (dotted lines). In A, ***P < 0.01 versus SKOV-3. In B, *P < 0.05 versus SKOV-3.

Exosomal protein release was expressed as total exosomal protein (*i.e.* particulate material with a buoyant density of 1.15 to 1.19 g/ml). OVCAR-3 cells released 0.44 ± 0.05 μg of exosomal protein per million cells per 24 h (*n* = 6; *i.e.* 6 different isolations from ~300 × 10^6^ cells each) whereas SKOV-3 cells released 1.22 ± 0.11 μg of exosomal protein per million cells per 24 h (*n =* 6; i.e. 6 different isolations from ~300 × 10^6^ cells each) (Figure 3A). SKOV-3 cells released significantly more exosomes (~2.7-folds) in 24 h compared to OVCAR-3 cells (p < 0.001). These results were confirmed using ELISA (see Methods). The number of exosome particles (NEP) released from SKOV-3 cells was ~2.2 fold higher than the release from OVACR-3 cells (8.07 × 10^8^ ± 5.9 × 10^7^ vs 1.63 × 10^9^ ± 3.04 ×10^8^ NEP in OVCAR-3 and SKOV-3, respectively, p < 0.05) (Figure 3B). In addition, NEP and exosomal protein pellet concentration were significant correlated (Spearman’s r = 0.58; p = 0.037).

### miRNA profile

**Figure 4:**
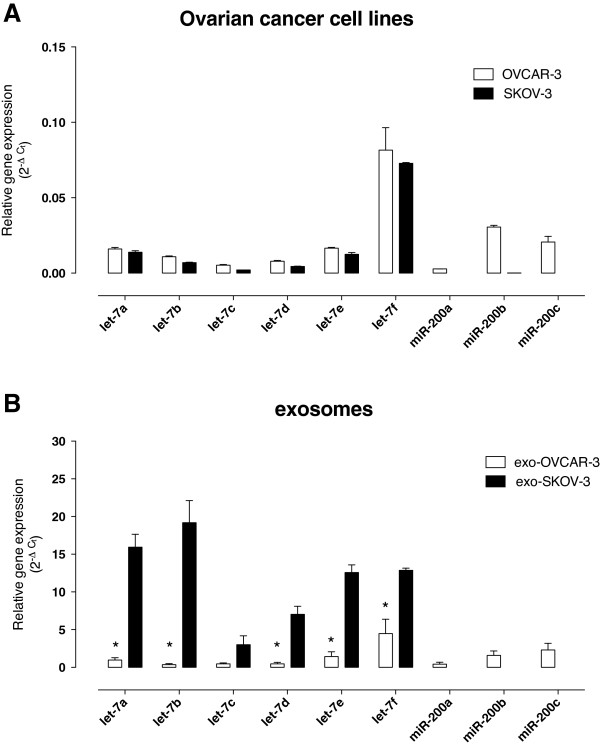
**Expression of let-7 and miR-200 families.** Total microRNA was extracted using the Ambion mirVana PARIS Kit (Invitrogen). Real-time PCR was performed with miScript SYBR Green Kit to compare let-7 and miR-200 family expression between ovarian cancer cell lines (OVAR-3 and SKOV-3 cells) **(A)** and between cell-derived exosomes (exo-OVAR-3 and exo-SKOV-3) **(B)**. Data are represented as mean ± SEM (*n* = 6). In **A** and **B**, ANOVA *p* < 0.0001 between groups. In **B**, *p < 0.001 vs exo-SKOV-3.

The presence and abundance of specific microRNA (let-7 and miR-200 family) in both OVCAR-3 and SKOV-3 cell-derived and exosome-derived were determined. The expression profile of miRNA (miR-let7 and miR-200) in the two cell lines was significantly different (ANOVA, p < 0.001). Post-hoc pairwise comparisons identified significant differences in the expression of miR-200 but not let-7 transcripts. (Bonferroni’s Multiple comparison test, p < 0.01, Figure 4A).

**Figure 5:**
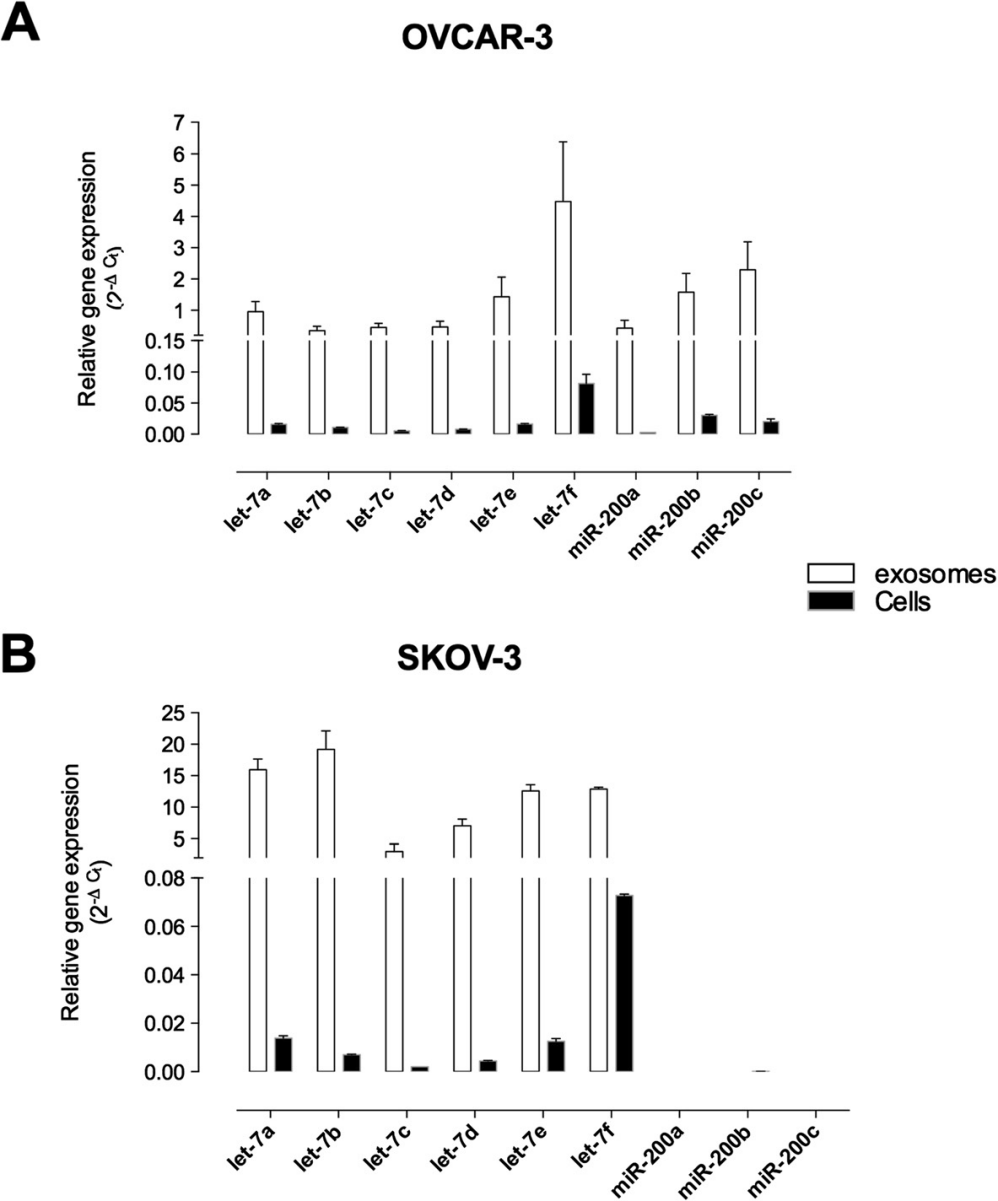
**Comparison of miRNA transcripts expression between cells and exosomes.** The miRNA expressions between cells and exosomes in OVCAR-3 **(A)** and SKOV-3 **(B)**. Data is represented by mean ± SEM (*n =* 6 per group).

In exosomes, miRNA expression profile (miR-let7 and miR-200) was significantly different between OVCAR-3- and SKOV-3-derived exosomes. exo-SKOV-3 expressed significantly more (*n =* 6, *p* < 0.001) let-7 transcripts compared to exo-OVCAR-3. Moreover, miR-200b and miR-200c were only expressed in exo-OVCAR-3 reflecting the miRNA signature in cells (Figure 4B). In addition, the expression of miRNA transcript was significantly higher in exosomes compared with the expression in cells (Figure 5A and 5B).

## Discussion

The aim of this study was to test the hypothesis that ovarian cancer cell invasiveness is associated with altered release of exosomes and discordant exosomal sequestration of miRNA. Two ovarian cancer cell lines of epithelial cell origin but with different invasive potentials were used to test this hypothesis. In this study, the release of exosomes from cells incubated under similar conditions, was ~2-folds greater from SKOV-3 cells (high invasive) than from OVCAR-3 cells (low invasive). Exosomes released by SKOV-3 cells contained a greater abundance of let-7 miRNA transcripts than OVCAR-3 cells. When exosomal miRNA content was compared with that of the cell of origin, SKOV-3 cell-derived exosomes displayed no correlation with the cell origin. In contrast, the miRNA profiles of OVCAR-3 cell-derived exosomes and their cells of origin were highly correlated. The data obtained established that the release and miRNA content of exosomes differs significantly between ovarian cancer cell lines and correlates with their invasive potential. Furthermore, the data are consistent with the hypothesis that selective packaging of specific miRNA transcripts into exosomes may represent a regulatory mechanism to maintain invasive phenotype by reducing the intracellular availability of miRNAs (*i.e.* let-7 miRNAs) with known tumor suppressor activity.

In this study, we initially confirmed that SKOV-3 have a greater capacity to invade than OVCAR-3 cells. Using a 3D *in vitro* culture model, we observed that SKOV-3 cells were 2–3 times more invasive than OVCAR-3 cells (p < 0.0001). These data are consistent with previous publications [17].

Exosomes released from ovarian cancer cells *in vitro* were isolated and characterised using well-established methods [9,18,19,21-23]. Exosomes isolated from both cell lines displayed characteristic morphology, buoyant density (1.15 to 1.19 g/ml) and exosomal markers (*i.e.* CD63 and CD9 - members of the tetraspanin family and one of the most abundant protein families present in exosomes [24]. Previously, we [19] and others [7,21,25] have successfully used such preparation of exosomes for both proteomic analysis and RNA profiling.

In this study, we defined the release of exosomes from ovarian cancer cells as the accumulation (in exosome-free incubation medium) of CD63 and CD9 positive, particulate protein with a density of 1.15 to 1.19 g/ml. Exosome release was cell type specific and correlated with cell invasiveness. When cells were incubated under identical conditions, the release of exosomes from SKOV-3 cells was more than 2-folds greater than that of OVCAR-3 cells (p < 0.001). Exosome have been previously isolated from both OVCAR-3 [7,26] and SKOV-3 cells [9], however, this is the first study to directly compare exosome release from these cell lines.

*In vivo*, exosomes released from ovarian tumours may be present in blood and other biological fluids. Previously, Taylor and Gercel-Taylor [9] isolated exosomes from serum samples of women with ovarian cancer. The concentration of exosomes was significantly elevated with disease progression. The release of exosomes has also been found to correlate with the invasiveness of other tumour cell lines [27]. Even though the molecular mechanism triggering the release of exosomes is poorly understood, factors promoting exosome release have been identified, including: hypoxia, anticancer drugs, thermal and oxidative stress [28-31]. It is well established that heterogeneity exists among tumour cells even in a single tumour [16].

This study further characterised ovarian cancer cell exosome content by RT-PCR (miRNA content). Of particular note was the variation in expressions of two miRNA families the let-7 and miR-200 transcripts. Iori, Visone *et al.*[16] performed a large scale miRNA profiling of ovarian cancer tissues. Let-7 was found to be down-regulated and miR-200 transcripts were among the most significantly up-regulated in epithelial ovarian cancer tissues compared to normal tissues [16]. Both let-7 and miR-200 families are tumour suppressors; let-7 repressing cell proliferation while miR-200 regulate epithelial to mesenchymal transition [32]. Taylor and Gercel-Taylor [9] has compared the expression of selected miRNA in exosomes and originating cells. Expression levels of most miRNA including let-7a, b, c, d, f and miR-200a, b, c have been found to be the same, whereas let-7e was elevated in exosomes compared to the cell, suggesting active selection during miRNA compartmentalisation into exosomes [28]. The overall expressions of let-7 transcripts were more abundant in OVCAR-3 cells than SKOV-3 cells. Interestingly, in exosomes, let-7 transcripts were more abundant in SKOV-3 cell derived exosomes. The low expression of let-7 in SKOV-3 cells may be due to the compartmentalisation into exosomes. Exosomes can either fuse with the lysosomes to degrade their contents or release them out of the cells to decrease the expression of tumour suppressor miRNAs, ultimately leading to their aggressive phenotype. The miR-200 transcripts were only present in exosomes from OVCAR-3 as SKOV-3 did not express miR-200 s in their cells. Previous studies have compared miRNA expression in normal and cancerous tissue or cell lines but have not compared expressions of different metastatic potentials.

Recently, we demonstrated that proteins are selectively packaged into placental cells exosomes by a process that, at least in part, is regulated by the extracellular milieu (*i.e.* low oxygen tension) [18,33]. In addition, Li *et al.,* (2013) [34] reported that microvesicles (*i.e*. exosomes) from glioma cells contain selective packaged miRNA that modify the gene expression in cell target [34]. These observations are consistent with data obtained in this study and the hypothesis that exosomal oligonucleotides are selective packaged into exosome by endosomal process (that are yet to be identified).

In this study, the expression of let-7 miRNA transcripts was similar in both parent cell lines, however, miR-200 transcripts were only identified in OVCAR-3 cells. In contrast, exosomal expression of both let-7 and 200 miRNA transcripts displayed a dramatic cell specific variation. The expression of let-7 miRNA transcripts in exosomes released from SKOV-3 cells was 7–20 times greater than in exosomes released from OVCAR-3 cells. Similar, to the expression profile in the parent cells, miR-200 transcripts were only identified in exosomes from OVCAR-3. These data are suggestive that ovarian cancer cell invasiveness is not affected by the expression of let-7 miRNA transcripts but that repression of miR-200 transcripts is associated with increased invasive capacity. Furthermore, the data obtained establish the selective and cell specific packaging of miRNA transcripts into exosomes.

The expression of miRNA-200 transcripts in OVCAR-3 cells and their absence in the more aggressive SKOV-3 cell line is consistent with the proposed involvement of miR-200 s in tumor suppression via the inhibition of epithelial-mesenchymal transition (EMT), the initiating step of metastasis [32]. The expression of miR-200 s in OVCAR-3 may be a contributing factor in determining their lower invasive potential when compared to SKOV-3 cells. In support of this suggestion, the let-7 miRNA transcripts a-f were identified in both cell types but were more abundant in OVCAR-3.

## Conclusions

**Figure 6:**
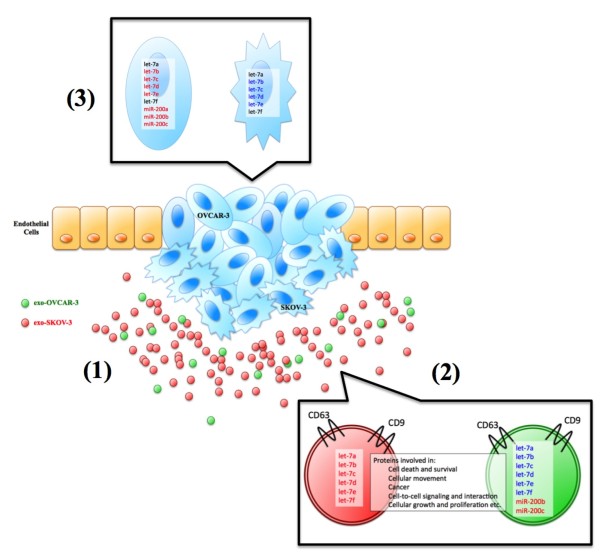
**Proposed model of ovarian cancer cell-derived exosome action.** Tumour cells with different invasion capacity exist in a single epithelial ovarian tumour, releasing exosomes. High invasive tumour cells (SKOV-3) release significantly more exosomes **(1)**, containing proteins involved in processes such as: cell death and survival, cellular movement, cancer, cell-to-cell signalling and interaction, cellular growth and proliferation. The let-7 family that are known to suppress cell proliferation was significantly more expressed in exosomes from high invasive exosomes (from SKOV-3). On the other hand, the miR-200 family that suppress epithelial to mesenchymal transition was only expressed in exosomes derived from low invasive cells (from OVCAR-3) **(2)**. The miRNA profile differs between exosomes derived from cells with different invasive capacity (OVAR-3 versus SKOV-3) **(3)**.

Based on the data obtained in this study and on previously published observations, we propose the following model of ovarian cancer cell exosome release and action (Figure 6). Tumour cells with different invasion capacity exist in a single epithelial ovarian tumour, releasing exosomes. High invasive tumour cells (SKOV-3) release significantly more exosomes [1], containing proteins involved in processes such as: cell death and survival, cellular movement, cancer, cell-to-cell signalling and interaction, cellular growth and proliferation. The let-7 family that is known to suppress cell proliferation was significantly more expressed in exosomes from high invasive exosomes (from SKOV-3). On the other hand, the miR-200 family that suppresses epithelial to mesenchymal transition was only expressed in exosomes derived from low invasive cells (from OVCAR-3) [2]. The miRNA profile differs between exosomes derived from cells with different invasive capacity (OVCAR-3 versus SKOV-3) (3). Interestingly, significantly less let-7 family miRNA was expressed in the high invasive cells (SKOV-3) although it was highly expressed in exosomes. Within the tumour, high invasive tumour cell derived-exosomes signal low invasive tumour cells and low invasive tumour cell derived-exosomes can signal high invasive tumour cells to increase invasion of recipient cells.

## Competing interests

The authors declare that they have no competing interests.

## Author’s contributions

MK and CS contributed in generating experimental data. MK, CS, JT, SEI, MDM, and GER contributed in discussion and reviewed/edited manuscript. MK, CS and GER wrote the manuscript and drew the figures. All authors read and approved the final manuscript.
